# Causability and explainability of artificial intelligence in medicine

**DOI:** 10.1002/widm.1312

**Published:** 2019-04-02

**Authors:** Andreas Holzinger, Georg Langs, Helmut Denk, Kurt Zatloukal, Heimo Müller

**Affiliations:** ^1^ Institute for Medical Informatics, Statistics and Documentation Medical University Graz Graz Austria; ^2^ Department of Biomedical Imaging and Image‐guided Therapy Computational Imaging Research Lab, Medical University of Vienna Vienna Austria; ^3^ Institute of Pathology Medical University Graz Graz Austria

**Keywords:** artificial intelligence, causability, explainability, explainable AI, histopathology, medicine

## Abstract

Explainable artificial intelligence (AI) is attracting much interest in medicine. Technically, the problem of explainability is as old as AI itself and classic AI represented comprehensible retraceable approaches. However, their weakness was in dealing with uncertainties of the real world. Through the introduction of probabilistic learning, applications became increasingly successful, but increasingly opaque. Explainable AI deals with the implementation of transparency and traceability of statistical black‐box machine learning methods, particularly deep learning (DL). We argue that there is a need to go beyond explainable AI. To reach a level of *explainable medicine* we need causability. In the same way that usability encompasses measurements for the quality of use, causability encompasses measurements for the quality of explanations. In this article, we provide some necessary definitions to discriminate between explainability and causability as well as a use‐case of DL interpretation and of human explanation in histopathology. The main contribution of this article is the notion of causability, which is differentiated from explainability in that causability is a property of a person, while explainability is a property of a system

This article is categorized under:

Fundamental Concepts of Data and Knowledge > Human Centricity and User Interaction

## Graphical abstract

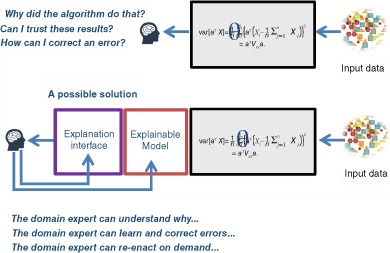

## 1 INTRODUCTION AND MOTIVATION

*Artificial intelligence (AI)* is perhaps the oldest field of computer science and very broad, dealing with all aspects of mimicking cognitive functions for real‐world problem solving and building systems that learn and think like people. Therefore, it is often called machine intelligence (Poole, Mackworth, & Goebel, 1998) to contrast it to human intelligence (Russell & Norvig, 2010). The field revolved around the intersection of cognitive science and computer science (Tenenbaum, Kemp, Griffiths, & Goodman, 2011). AI now raises enormous interest due to the practical successes in machine learning (ML). In AI there was always a strong linkage to explainability, and an early example is the Advice Taker proposed by McCarthy in 1958 as a “program with common sense” (McCarthy, 1960). It was probably the first time proposing common sense reasoning abilities as the *key* to AI. Recent research emphasizes more and more that AI systems should be able to build causal models of the world that support explanation and understanding, rather than merely solving pattern recognition problems (Lake, Ullman, Tenenbaum, & Gershman, 2017).

ML is a very practical field of AI with the aim to develop software that can automatically learn from previous data to gain knowledge from experience and to gradually improve it's learning behavior to make predictions based on new data (Michalski, Carbonell, & Mitchell, 1984). The grand challenges are in sense‐making, in context understanding, and in decision making under uncertainty (Holzinger, 2017). ML can be seen as the workhorse of AI and the adoption of data intensive ML methods can meanwhile be found everywhere, throughout science, engineering and business, leading to more evidence‐based decision‐making (Jordan & Mitchell, 2015). The enormous progress in ML has been driven by the development of new statistical learning algorithms along with the availability of large data sets and low‐cost computation (Abadi et al., 2016). One nowadays extremely popular method is deep learning (DL).

DL is a family of ML models based on deep convolutional neural networks having a long history (Schmidhuber, 2015). DL is very popular today because they are achieving amazing results even at human level performance (LeCun, Bengio, & Hinton, 2015). A best practice example is a recent work of the Thrun group, where they achieved with a DL approach performance on par with medical doctors, demonstrating that such approaches are able to classify skin cancer with a level of competence comparable to human dermatologists (Esteva et al., 2017). A further example is the promising results of identifying diabetic retinopathy and related eye diseases (Ting et al., 2017). All these are very good examples of the progress and usefulness of AI, but even the most prominent proponents of these (automatic) approaches recently emphasize that *usable intelligence* is difficult to reach because we need not only to learn from prior data, to extract knowledge, to generalize, and to fight the curse of dimensionality, but to disentangle the underlying explanatory factors of the data in order to understand the context in an application domain (Bengio, Courville, & Vincent, 2013), where to date a doctor‐in‐the‐loop is indispensable (Holzinger, 2016).

*Medicine* as application domain is among the greatest challenges of AI/ML/DL. In medical decision support we are confronted with uncertainty, with probabilistic, unknown, incomplete, imbalanced, heterogeneous, noisy, dirty, erroneous, inaccurate and missing data sets in arbitrarily high‐dimensional spaces (Holzinger, Dehmer, & Jurisica, 2014), (Lee & Holzinger, 2016). Often we are simply lacking of large data sets (Holzinger, 2016). A grand goal of future medicine is in modeling the complexity of patients to tailor medical decisions, health practices and therapies to the individual patient (Holzinger, 2014). This poses challenges particularly in the integration, fusion and mapping of various distributed and heterogeneous data up to the visual analysis of these heterogeneous data (Turkay, Jeanquartier, Holzinger, & Hauser, 2014). Consequently, *explainable‐AI* in the context of medicine must take into account that diverse data may contribute to a *relevant* result. This requires that medical professionals must have a possibility *to understand how and why* a machine decision has been made (Holzinger, Biemann, Pattichis, & Kell, 2017).

*Explainability* is at least as old as AI itself and rather a problem that has been caused by it. In the pioneering days of AI (Newell, Shaw, & Simon, 1958), reasoning methods were logical and symbolic. These approaches were successful, but only in a very limited domain space and with extremely limited practical applicability. A typical example is MYCIN (Shortliffe & Buchanan, 1975), which was an expert system developed in Lisp to identify bacteria causing severe infections and to recommend antibiotics. MYCIN was never used in clinical routine, maybe because of its stand‐alone character and the high effort in maintaining its knowledge base. However, these early AI systems reasoned by performing some form of logical inference on human readable symbols, and were able to provide *a trace of their inference steps*. This was the basis for explanation, and there is some early related work available, for example, (Johnson, 1994; Lacave & Diez, 2002; Swartout, Paris, & Moore, 1991). Here, we should mention that there are three types of explanations: (1) a peer‐to‐peer explanation as it is carried out among physicians during medical reporting; (2) an educational explanation as it is carried out between teachers and students; (3) A scientific explanation in the strict sense of science theory (Popper, 1935). We emphasize that in this article we mean the first type of explanation.

In medicine there is growing demand for AI approaches, which are not only performing well, but are trustworthy, transparent, interpretable and explainable for a human expert; in medicine, for example, sentences of natural language (Hudec, Bednrov, & Holzinger, 2018). Methods and models are necessary to reenact the machine decision‐making process, to reproduce and to comprehend both the learning and knowledge extraction process. This is important, because for decision support it is necessary to understand the **causality** of learned representations (Gershman, Horvitz, & Tenenbaum, 2015; Pearl, 2009; Peters, Janzing, & Schölkopf, 2017).

Moreover, explainability of AI could help to enhance trust of medical professionals in future AI systems. Research towards building explainable‐AI systems for application in medicine requires to maintain a high level of learning performance for a range of ML and human‐computer interaction techniques. There is an inherent tension between ML performance (predictive accuracy) and explainability. Often the best‐performing methods such as DL are the least transparent, and the ones providing a clear explanation (e.g., decision trees) are less accurate (Bologna & Hayashi, 2017).

Currently, explanations of why predictions are made, or how model parameters capture underlying biological mechanisms are elusive. A further constraint is that humans are limited to visual assessment or review of explanations for a (large) number of axioms. This result in one of the main question: Can we deduce properties without experiments—directly from pure observations? (Peters et al., 2017).

Understanding, interpreting, or explaining are often used synonymously in the context of explainable‐AI (Doran, Schulz, & Besold, 2017), and various techniques of interpretation have been applied in the past. There is a helpful discussion on the “Myth of model interpretability” by Lipton (2016). In the context of explainable‐AI the term “understanding” usually means a *functional understanding* of the model, in contrast to a low‐level algorithmic understanding of it, that is, to seek to characterize the model's black‐box behavior, without trying to elucidate its inner workings or its internal representations. Montavon, Samek, and Müller (2017) discriminate in their work between *interpretation*, which they define as a mapping of an abstract concept into a domain that the human expert can perceive and comprehend; and *explanation*, which they define as a collection of features of the interpretable domain, that have contributed to a given example to produce a decision.

We argue that in medicine explainable AI is urgently needed for many purposes including medical education, research and clinical decision making (Holzinger, 2018). If medical professionals are complemented by sophisticated AI systems and in some cases future AI systems even play a huge part in the decision making process, human experts must still have the means—on demand—to understand and to retrace the machine decision process.

At the same time, it is interesting to know that while it is often assumed that humans are always able to explain their decisions, this is often *not* the case! Sometimes experts are not able to provide an explanation based on the various heterogeneous and vast sources of different information. Consequently, explainable‐AI calls for confidence, safety, security, privacy, ethics, fairness and trust (Kieseberg, Weippl, & Holzinger, 2016), and brings usability (Holzinger, 2005) and *Human‐AI Interaction* into a new and important focus (Miller, Howe, & Sonenberg, 2017). All these aspects together are crucial for applicability in medicine generally, and for future personalized medicine, in particular (Hamburg & Collins, 2010).

First we provide some definitions to explain what kind of explainability we mean—this will lead us to the term “Causability” in contrast to the well‐known term “Causality”; then we discuss briefly the state‐of‐the‐art of some current explainable models, and continue with an example and a medical use‐case from histopathology. We conclude with pointing to the urgent need of a systems causability scale to measure the quality of an explanation (Hoffman, Mueller, Klein, & Litman, 2018), which must also include social aspects of human communication (Miller, 2019).

## 2 FROM EXPLAINABILITY TO CAUSABILITY

In an ideal world both human and machine statements would be identical, and congruent with the ground truth, which is defined for machines and humans equally. However, in the real world we face two problems:

(i) Ground truth cannot always be well defined, especially when making a medical diagnosis.

(ii) Human (scientific) models are often based on causality as an ultimate aim for understanding underlying mechanisms, and while correlation is accepted as a basis for decisions, it is viewed as an intermediate step. In contrast today's successful ML algorithms are typically based on probabilistic models and provide only a crude basis for further establishing causal models. When discussing the explainability of a machine statement, we therefore propose to distinguish between:

**Table:** 

Explainability	in a technical sense highlights decision‐relevant parts of the used representations of the algorithms and active parts in the algorithmic model, that either contribute to the model accuracy on the training set, or to a specific prediction for one particular observation. It does not refer to an explicit human model.
Causability	as the extent to which an explanation of a statement to a human expert achieves a specified level of causal understanding with effectiveness, efficiency and satisfaction in a specified context of use.

As causability is measured in terms of effectiveness, efficiency, satisfaction related to causal understanding and its transparency for a user, it refers to a human understandable model. This is always possible for an explanation of a human statement, as the explanation is per se defined related to a human model. However, to measure the causability of an explanation of a machine statement this has to be based on a causal model, which is not the case for most ML algorithms, or a mapping between both has to be defined.

**Figure 1:**
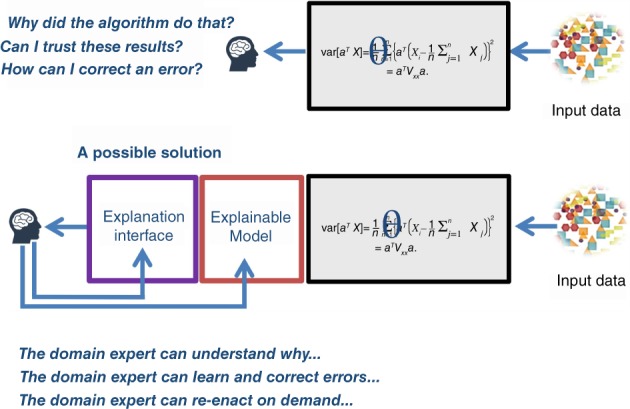
The best performing statistical approaches today are black‐boxes and do not foster understanding, trust and error correction (above). This implies an urgent need not only for explainable models, but also for explanation interfaces—and as a measure for the human‐AI interaction we need the concept of causability—analogous to usability in classic human‐computer interaction

Here, we must distinguish between an explainable model (“explainable AI”) and an explanation interface which makes the results gained in the explainable model not only usable but also useful to the expert. As a measure for the usability of such an Human‐AI interaction interface we propose to use the term causability (see Figure 1).

The term AI itself is actually an unfortunate one for engineering, since the phenomenon of intelligence is very difficult to define and is dependent on a wealth of different factors; Therefore, we limit ourselves here only to explicitly relevant facts for explainability.

Understanding is not only recognizing, perceiving and reproducing (stimulus–response on a physiological level), and not only the content comprehension and mere representation of facts, but the intellectual understanding of the context in which these facts appear. Rather, understanding can be seen as a bridge between perceiving and reasoning. From capturing the context, without doubt an important indicator of intelligence, the current state‐of‐the‐art AI is still many miles away. On the other hand, people are very well able to instantaneously capture the context and make very good generalizations from very few data points.

Explaining (Interpretation) means to provide causes of observed phenomena in a comprehensible manner through a linguistic description of its logical and causal relationships. In the theory of science, according to the hypothetical‐deductive model of Karl Popper, causal explanations are the foundation of science in order to derive facts from laws and conditions in a deductive way. Consequently, causality and causal reasoning is an extremely important area for explainable AI (Pearl & Mackenzie, 2018). Understanding and explaining are prerequisites for retraceability. The question remains open: “What is principally understandable for a human?”.

Directly understandable, hence explainable for humans are data, objects or any graphical representations ≤ℝ^3^, for example, images (arrays of pixels, glyphs, correlation functions, graphs, 2D/3D projections etc., or text (sequences of natural language). Humans are able to perceive data as images or words and process it as information in a physiological sense, cognitively interpret the extracted information with reference to their subjective previous knowledge (humans have a lot of prior knowledge) and integrating this new knowledge into their own cognitive knowledge space. Strictly speaking, there must be made a distinction between understanding natural images (pictures), understanding text (symbols) and understanding spoken language.

Not directly understandable, thus not explainable for humans are abstract vectorspaces >ℝ^3^(e.g., word‐embeddings) or undocumented, that is, previously unknown input features (e.g., sequences of text with unknown symbols (e.g., Chinese for an English speaker). An example shall illustrate it: in the so‐called word embedding (Mikolov, Chen, Corrado, & Dean, 2013), words and/or phrases are assigned to vectors. Conceptually, this is a mathematical embedding of a space with one dimension per word into a continuous vector space with a reduced dimension. Methods to generate such a “mapping” include, for example, deep neural nets and probabilistic models with an explicit representation in relation to the context in which the words appear.

For more details on the theory behind scientific explainability we refer to the principles of abductive reasoning (Ma et al., 2010) and point to some current work (Babiker & Goebel, 2017; Goebel et al., 2018).

## 3 GENERAL APPROACHES OF EXPLAINABLE AI MODELS

We can distinguish two types of explainable AI, which can be denominated with Latin names used in law (Fellmeth & Horwitz, 2009): posthoc explainability = “(lat.) after this”, occurring after the event in question; for example, explaining what the model predicts in terms of what is readily interpretable; ante‐hoc explainability = “(lat.) before this”, occurring before the event in question; for example, incorporating explainability directly into the structure of an AI‐model, explainability by design.

**Posthoc systems** aim to provide local explanations for a specific decision and make it reproducible on demand (instead of explaining the whole systems behavior). A representative example is local interpretable model‐agnostic explanations (LIME) developed by Ribeiro, Singh, and Guestrin (2016b), which is a **model‐agnostic** system, where *x* ∈ ℝ^*d*^ is the original representation of an instance being explained, and *x*^′^ ∈ ℝ^*d*′^ is used to denote a vector for its interpretable representation (e.g., *x* may be a feature vector containing word embeddings, with *x*^′^ being the bag of words). The goal is to identify an interpretable model over the *interpretable representation* that is **locally faithful** to the classifier. The explanation model is *g* : ℝ^*d*′^ → ℝ, *g* ∈ *G*, where *G* is a class of potentially interpretable models, such as linear models, decision trees, or rule lists; given a model *g* ∈ *G*, it can be visualized as an explanation to the human expert (for details please refer to (Ribeiro, Singh, & Guestrin, 2016a)). Another example for a posthoc system is black box explanations through transparent approximations (BETA), a model‐agnostic framework for explaining the behavior of any black‐box classifier by simultaneously optimizing for fidelity to the original model and interpretability of the explanation introduced by Lakkaraju, Kamar, Caruana, and Leskovec (2017).

Bach et al. (2015) presented a general solution to the problem of understanding classification decisions by pixel‐wise decomposition of nonlinear classifiers which allows visualization of the contributions of single pixels to predictions for kernel‐based classifiers over bag of words features and for multilayered neural networks.

**Ante‐hoc systems** are interpretable by design towards glass‐box approaches (Holzinger et al., 2017, 2018); typical examples include linear regression, decision trees and fuzzy inference systems. The latter have a long tradition and can be designed from expert knowledge or from data and provides—from the viewpoint of Human‐AI interaction—a good framework for the interaction between human expert knowledge and hidden knowledge in the data (Guillaume, 2001). A further example was presented by Caruana et al. (2015), where high‐performance generalized additive models with pairwise interactions (GAMs) were applied to problems from the medical domain yielding intelligible models, which uncovered surprising patterns in the data that previously had prevented complex learned models from being fielded in this domain; of importance is that they demonstrated *scalability* of such methods to large data sets containing hundreds of thousands of patients and thousands of attributes while remaining intelligible and providing accuracy comparable to the best (unintelligible) ML methods. A further example for ante‐hoc methods can be seen in Poulin et al. (2006), where they described a framework for visually explaining the decisions of any classifier that is formulated as an additive model and showed how to implement this framework in the context of three models: naïve Bayes, linear support vector machines and logistic regression, which they implemented successfully into a bioinformatics application (Szafron et al., 2004).

### 3.1 Example: interpreting a deep neural network

Deep neural networks (DNN), particularly convolutional neural networks (CNN) and recurrent neural networks (RNN) have been demonstrated to be applicable to a wide range of practical problems, from image recognition (Simonyan & Zisserman, 2014) and image classification (Esteva et al., 2017) to movement recognition (Singh et al., 2017). At the same time these approaches are also remarkable from a scientific point of view, since they reflect human processes. For instance, humans organize their ideas hierarchically (Bengio, 2009; Schmidhuber, 2015), and recent work has observed evidence about how learned models in CNNs are similar to those found in the human visual ventral pathway (Khaligh‐Razavi & Kriegeskorte, 2014). Since the early phases of research on artificial neural networks, people have tried to make them explainable. One of the early approaches was the approach of gradients in the form of sensitivity analysis (Simonyan & Zisserman, 2014).

An artificial neural network (NN) is a collection of neurons organized in a sequence of multiple layers, where neurons receive as input the neuron activations from the previous layer, and perform a simple computation (e.g., a weighted sum of the input followed by a nonlinear activation). The neurons of the network jointly implement a complex nonlinear mapping from the input to the output. This mapping is learned from the data by adapting the weights of each individual neuron using backpropagation, which repeatedly adjusts the weights of the connections in the network in order to minimize the difference between the current output vector and the desired output vector. As a result of the weight adjustments, internal hidden units which are not part of the input or output come to represent important features of the task domain, and the regularities in the task are captured by the interactions of these units (refer to the original paper of Rumelhart, Hinton, and Williams (1986) and the review by Widrow and Lehr (1990) for an overview).

Typically, deep neural networks are trained using supervised learning on large and carefully annotated data sets. However, the need for such data sets restricts the space of problems that can be addressed. On one hand, this has led to a proliferation of deep learning results on the same tasks using the same well‐known data sets (Rolnick, Veit, Belongie, & Shavit, 2017). On the other hand, to the emerging relevance of weakly‐ and un‐supervised approaches that aim at reducing the need for annotations (Schlegl, Seeböck, Waldstein, Schmidt‐Erfurth, & Langs, 2017; Seeböck et al., 2018).

Several approaches to probe and interpret deep neural networks exist (Kendall & Gal, 2017). ***Uncertainty*** provides a measure of how small perturbations of training data would change model parameters, the so‐called *model uncertainty* or *epistemic uncertainty*, or how input parameter changes would affect the prediction for one particular example, the *predictive uncertainty*, or *aleatoric variability* (Gal, 2016). In a *Bayesian Deep Learning* approach, Pawlowski, Brock, Lee, Rajchl, and Glocker (2017) approximate model parameters through variational methods, resulting in uncertainty information of model weights, and a means to derive *predictive uncertainty* from the model outputs. Providing uncertainty facilitates the appropriate use of model predictions in scenarios where different sources of information are combined as typically the case in medicine. We can further differentiate aleatoric uncertainty, into *homoscedatic* uncertainty independent of a particular input, and *heteroscedatic* uncertainty possibly changing with different inputs to the system.

Methods for ***attribution*** seek to link a particular output of the deep neural network to input variables. Sundararajan, Taly, and Yan (2017) analyze the gradients of the output when changing individual input variables. In a sense this traces the prediction uncertainty back to the components of a multivariate input. Zhou, Khosla, Lapedriza, Oliva, and Torralba (2016) use activation maps to identify parts of images relevant for a network prediction. Recently attribution approaches for generative models have been introduced. Baumgartner, Koch, Tezcan, Ang, and Konukoglu (2017) demonstrate how image areas that are specific to the foreground class in *Wasserstein Generative Adversarial Networks (WGAN)* can be identified and high‐lighted in the data. Biffi et al. (2018) learn interpretable features for variational auto encoders (VAE) by learning gradients in the latent embedding space that it linked to the classification result.

**Figure 2:**
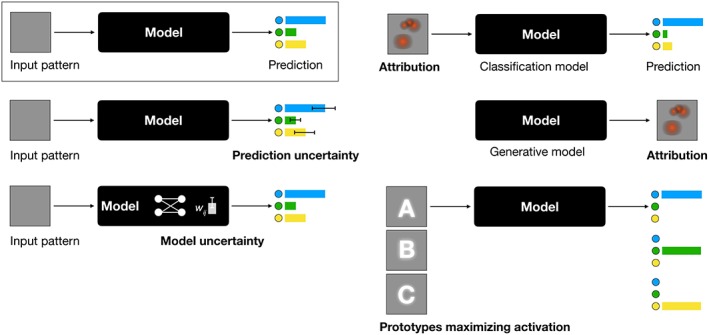
An overview of how deep learning models can be probed for information regarding uncertainty, attribution, and prototypes

***Activation maximization*** (Montavon et al., 2017) identifies input patterns that lead to maximal activations relating to specific classes in the output layer (Berkes & Wiskott, 2006; Simonyan & Zisserman, 2014). This makes the visualization of *prototypes* of classes possible, and assesses which properties the model captures for classes^1^ (Erhan, Bengio, Courville, & Vincent, 2009). For a neural network classifier mapping data points ***x*** to a set of classes (*ω*_*c*_)_*c*_, the approach identifies highly probable regions in the input space, that create high output probabilities for a particular class. These positions can be found by introducing a data density model in the standard objective function log*p*(*ω*_*c*_ | ***x***) − *λ*‖***x***‖^2^ that is maximized during model training. Instead of the ℓ_2_‐norm regularizer that implements a preference for inputs that are close to the origin, the density model or “expert” (Montavon et al., 2017) results in the term log*p*(*ω*_*c*_| ***x***) + log*p*(***x***) that is to be maximized. Here, the prototype is encouraged to simultaneously produce strong class response and to resemble the data. By application of Bayes' rule, the newly defined objective can be identified, up to modeling errors and a constant term, as the class‐conditioned data density *p*(***x***| *ω*_*c*_). The learned prototype thus corresponds to the most likely input ***x*** for the class *ω*_*c*_ (Figure 2).

A possible choice for the expert is a Gaussian restricted Boltzmann machine (RBM). The RBM is a two‐layer, bipartite, undirected graphical model with a set of binary hidden units *p*(*h*), a set of (binary or real‐valued) visible units *p*(*v*), with symmetric connections between the two layers represented by a weight matrix *W*. The probabilistic semantics for an RBM is defined by its energy function (for details see the chapter by Hinton (2012). Its probability function can be written as: $\log p\left(x\right)=\sum_{j}f_{j}\left(x\right)-\frac{1}{2}x^{⊤}\Sigma^{-1}x+\mathrm{cst}.$, where $f_{j}\left(x\right)=\log\left(1+\exp\left(w_{j}^{⊤}x+b_{j}\right)\right)$ are factors with parameters learned from the data. When interpreting more complex concepts such as natural images classes, other density models such as convolutional RBM's (Lee, Grosse, Ranganath, & Ng, 2009) or pixel RNN's (Oord, Kalchbrenner, & Kavukcuoglu, 2016) are suitable.

The selection of the so‐called expert *p*(***x***) plays an important role. Basically, there are four different cases: In the case where “the expert” is absent, that is, the optimization problem reduces to the maximization of the class probability function *p*(*ω*_*c*_| ***x***). In the case where we see the other extreme, that is, the expert is overfitted on some data distribution, and thus, the optimization problem becomes essentially the maximization of the expert *p*(***x***) itself.

When using activation maximization for the purpose of model validation, an overfitted expert must be especially avoided, as the latter could hide interesting failure modes of the model *p*(*ω*_*c*_| ***x***). A slightly underfitted expert (case b), for example, that simply favors images with natural colors, can already be sufficient. On the other hand, when using AM to gain knowledge on a correctly predicted concept *ω*_*c*_, the focus should be to prevent underfitting. Indeed, an underfitted expert would expose optima of *p*(*ω*_*c*_| ***x***) potentially distant from the data, and therefore, the prototype ***x***^⋆^ would not be truly representative of *ω*_*c*_.

*Unsupervised learning and generative models*. In certain applications, it is helpful to not only predict based on input data, but learn the structure of a set of training examples, to either provide a parametric representation of its density *p*(*x*), or at least be able to sample from this density generating examples *of the same type* as the training examples. Examples are Boltzmann machines, autoencoders, or generative adversarial networks) which do not provide the density function directly, but are able to sample from it, usually via the following two steps:

1. Sample from a simple distribution $q\left(z\right)∼N\left(0,I\right)$ which is defined in an abstract code space Z;
2. Apply to the sample a decoding function g:Z→X, that maps it back to the original input domain.

There are two aspects of models learned by unsupervised learning that are relevant in the context of explainability. First, the latent representations learned in these models can hold structure that reflects relatively complex relationship patterns in the data. For instance, in Mikolov et al. (2013) the authors show that word embeddings can reflect semantic similarity. Second, being able to generate instances, or even instances that are *as close as possible* to an observation, provides means to study the difference of examples to a class. This is relevant in medicine, where the discovery and study of anomalies that are potentially linked to disease is relevant Schlegl et al. (2017).

One example is the *generative adversarial network (GAN)* introduced by Goodfellow et al. (2014). It consists of two models: a generative model *G* that captures the data distribution, and a discriminative model *D* that estimates the probability that a sample came from the training data rather than from *G*. The training procedure for *G* is to maximize the probability of *D* making an error—which works like a minimax (minimizing a possible loss for a worst case maximum loss) two‐player game. In the space of arbitrary functions *G* and *D*, a unique solution exists, with *G* recovering the training data distribution and *D* equal to $\frac{1}{2}$ everywhere; in the case where *G* and *D* are defined by multilayer perceptrons, the entire system can be trained with backpropagation.

To learn the generator's distribution *p*_*g*_ over data ***x***, a prior must be defined on the input noise variables *p*_***z***_(***z***), and then a mapping to the data space as *G*(***z***; *θ*_*g*_), where *G* is a differentiable function represented by a multilayer perceptron with parameters *θ*_*g*_. The second multilayer perceptron *D*(***x***; *θ*_*d*_) outputs a single scalar. *D*(***x***) represents the probability that ***x*** came from the data rather than *p*_*g*_. *D* can be trained to maximize the probability of assigning the correct label to both training examples and samples from *G*. Simultaneously *G* can be trained to minimize log(1 − *D*(*G*(***z***))); in other words, *D* and *G* play the following two‐player minimax game with value function *V*(*G*, *D*):

(1) $$\min_{G}\max_{D}V\left(D,G\right)=E_{x∼p_{\text{data}}\left(x\right)}\left[\log D\left(x\right)\right]+E_{z∼p_{z}\left(z\right)}\left[\log\left(1-D\left(G\left(z\right)\right)\right)\right].$$

Nguyen, Dosovitskiy, Yosinski, Brox, and Clune (2016) proposed building a prototype for *ω*_*c*_ by incorporating such a generative model in the activation maximization framework. The optimization problem is redefined as:

(2) $$\max_{z\in Z}\log p\left(\omega_{c}|g\left(z\right)\right)-\lambda {\left\|z\right\|}^{2},$$

where the first term is a composition of the newly introduced decoder and the original classifier, and where the second term is an ℓ_2_‐norm regularizer in the code space. Once a solution ***z***^⋆^ to the optimization problem is found, the prototype for *ω*_*c*_ is obtained by decoding the solution, that is, ***x***^⋆^ = *g*(***z***^⋆^).

The ℓ_2_‐norm regularizer in the input space can be understood in the context of image data as favoring gray‐looking images. The effect of the ℓ_2_‐norm regularizer in the code space can instead be understood as encouraging codes that have high probability. High probability codes do not necessarily map to high density regions of the input space; for more details refer to the excellent tutorial given by Montavon et al. (2017).

### 3.2 Example use‐case histopathology

This section demonstrates the complexity of explanations of a human pathologist. For the following diagnosis

*Steatohepatitis with mild portal and incomplete‐septal fibrosis and mild centrilobular fibrosis of chicken wire type. The morphological picture corresponds to alcoholic steatohepatitis*. History of alcohol abuse?

#### 3.2.1 Example for human posthoc explanation

**Figure 3:**
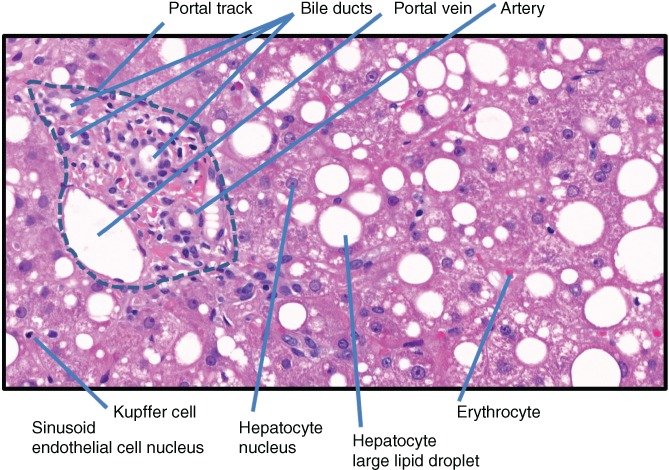
Features in a histology slide annotated by a human expert pathologist

We asked an experienced pathologist to explain what he considered *relevant* in the histology slides. A very small portion of the histological sections are shown in Figure 3 as illustration. For this specific diagnosis the pathologist gave the following facts as posthoc explanation:

- Liver biopsy with 10 evaluable portal fields.
- Lobule architecture preserved.
- Liver cells arranged in regular plates one cell layer thick.
- Portal fields slightly widened and slightly fibrotic.
- Isolated incomplete porto‐portal and porto‐central septa.
- Portal fields slightly inflamed with mixed‐cell (lymphocytes, sporadic neutrophil granulocytes) inflammatory inflitrates. Inflammation restricted to portal field.
- Parenchymatous border plate intact, liver cells with low anisocaryosis, moderately large droplet fatty liver (estimated parenchyma fatty degeneration at 30% of parenchymal area).
- Lobular central hepatic cells balloonized, light cytoplasmic with incorporation of Mallory‐Denk bodies.
- Most of these liver cells are surrounded by neutrophil granulocytes and some of them are interspersed (satelliteosis).
- Minor perivenular fibrosis (=central sclerosis).
- Kupffer cells slightly diffusely increased, isolated Kupffer cell nodules detectable.
- In the Berliner blue stain minor parenchymatous and Kupffer cell siderosis.

#### 3.2.2 Example for the ante‐hoc structure of explanations

We also asked the pathologist to explain the process and most relevant concepts in liver pathology. Please note, that the following description just demonstrates the structure and complexity and is far away from a textbook on liver pathology. The pathologist described the following procedure and observation as an ante‐hoc explanation for liver pathology:

- Describe in the macroscopic evaluation of the histological section the following features:
  –type, number and size of specimens (surgical specimen or biopsy)
  –tissue cohesion of biopsies
  –staining quality (H & E/CAB/Sirius red/iron/ev. PAS, immunohsistochemistry)
  –already visible exogenous tissue (tumor)
- Describe in microscopic evaluation at low magnification the following features:
  –lobular architecture preserved/disturbed in the sense of fibrosis or necrosis/destroyed in the context of cirrhosis or tumors
  –number of assessable portal fields (optimal 10–15 per slide)
  –liver cell (hepatocyte) plates regular‐one cell layer thick/several cell layers thick
  –inflammatory changes portal/lobular/combined; necrosis lobular peripheral/lobular central)
  –presence or absence of tissue
- Describe in microscopic evaluation at higher magnification the following features:
  –portal tracts: regular/extended/fibrotic/rounded/edematous
  –connective tissue parenchyma border: sharp/unsharp
  –parenchymatous border plate: preserved/partially destroyed/mostly destroyed/nonexistent inflammatory infiltrates portal/periportal‐interface, sparse/tight/localized‐follicular/ lymphocytic/ lymphohistiocytic/neutrophil‐granulocytic/ stressed‐eosinophil‐granulocytic/granulomatous;
  –abnormal content of the portal field not present (tumor cells/foreign bodies/parasites)
  –portal vessels (arteria hepatica, vena portae, and lymphatic vessels) present/expanded/narrowed/inflammatory;
  –Bile ducts: present/elongated/absent/single‐layer epithelium/multilayer epithelium/polymorphic epithelium/inflammatory changes/partially destructed/scarred/content (bile thrombus/porphyrinthrombus);
  –ductal reaction absent/low/pronounced /ductal cholestasis.
  –lobules (lobulus, liver parenchyma): Liver cells large/balloonized/small‐atrophic/anisocytosis/apoptosis
  –cytoplasm: granular/net‐like/light cytoplasmic‐glycogen‐rich/diffuse homogenized/focally homogenized
  –cytoplasmic inclusions fat large droplet/fat small droplet/ lipofuscin granules/siderin granules/AAT inclusions/Fibrinogen inclusions/Mallory Denk bodies (MDB), Hyaline bodies/bilirubin
  –canalicular bilirubinostasis
  –Necroses disseminated/confluent/lobular central/lobular periphery/bridging porto‐central/bridging centro‐central/massive; —liver cell nuclei anisocaryosis/pycnosis/punch cores/“sand cores“/core inclusions;
  –Kupffer cells focally increased (nodular)/diffus increased/enlarged/inclusions (siderin, pigment, erythrocytes, pathogen, foreign material);
  –star cells (stellate cells) increased
  –sinusoidal dilated/abnormal content (e.g., blood, fibrin, and tumor cells)
  –central vein lumen open/narrowed/ obliterated/inflamed/wall fibrosis.
  –fibrosis: portal/perisinusoidal/pericellular/perivenular/septal/porto‐portal/porto‐central/centro‐central/meshed wire fibrosis/incomplete cirrhosis/cirrhosis.
  –foreign tissue (tumor tissue to be characterized morphologically, primary/secondary‐metastatic/unclear).

For a specific case values of all above features contribute to the diagnosis with different weights and causal relations present in the human model on liver pathology, which an expert acquired by training and experience.

## 4 FUTURE OUTLOOK

### 4.1 Weakly supervised learning

Supervised learning is very expensive in the medical domain because it is cumbersome to get strong supervision information and fully ground‐truth labels. Particularly, labeling a histopathological image is not only time‐consuming but also a critical task for cancer diagnosis, as it is clinically important to segment the cancer tissues and cluster them into various classes (Xu, Zhu, Chang, Lai, & Tu, 2014). Digital pathological images generally have some issues to be considered, including the very large image size (and the involved problems for DL), insufficiently labeled images (the small training data available), the time needed from the pathologist (expensive labeling), insufficient labels (region of interest), different levels of magnification (resulting in different levels of information), color variation and artifacts (sliced and placed on glass slides) etc. (Komura & Ishikawa, 2018).

Weakly supervised learning (Xu et al., 2014) is an umbrella term for a variety of methods to construct predictive models by learning with weak supervision; weak because of either incomplete, inexact or inaccurate supervision. In a strong supervision task we want to learn *f* : *X* → *Y* from the training data set *D* = (***x***_**1**_, *y*_1_), … (***x***_**m**_, *y*_*m*_), wherein *X* is the feature space and (*x*_*i*_, *y*_*i*_) are always assumed to be identically and independently distributed data (which is not the case in real‐world problems!).

In the context of weakly supervised learning, we propose classifying whole slide images according to widely used scoring systems based on association with histomorphological characteristics and an overall predictive score, and to provide in addition a relevance map generated by observing the human expert during diagnosis making. By the combination of well‐known human features and new multiscale morphological classifiers the human causal model can be on the one hand extended and on the other hand the CNN model can be explained with known histomorphological features. We propose to extract from both benign and malign single cell nuclei and to classify chromatin organization (Araujo et al., 2017) within the nuclei to correlate these to histopathological features and molecular markers.

### 4.2 Structural causal models

A very important direction is research towards structural causal models (Pearl, 2009; Pearl & Mackenzie, 2018; Peters et al., 2017). Current AI work on either a statistical or model‐free mode. This entails severe limits on both effectiveness and performance. Such systems cannot reason about interventions and retrospection and, therefore, cannot serve for strong AI (Pearl & Mackenzie, 2018). To achieve human level intelligence, AI need the guidance of a model of reality, similar to the ones used in causal inference tasks. Consequently, we propose to: (1) develop new visualization techniques that can be trained by medical experts, as they can explore the underlying explanatory factors of the data and (2) formalize a structural causal model of human decision making and mapping features in these to DL approaches. In digital pathology such mechanistic models can be used to analyze and predict the response of a functional network behavior to features in histology slides, molecular data and family history.

### 4.3 Develop causability as a new scientific field

The human‐computer interaction community has established a range of usability methods (Holzinger, 2005). Similar to these usability methodologies, methods and tests, we need the development of causability methodologies, methods and tests, which are based on clear scientific principles and theories of causality in order to establish causability as a scientific field which will become necessary with increased use of AI. The same as usability measures ensures the “quality of use” (Bevan, 1995), causability measures must ensure the “quality of explanations”.

According to the three Layer Causal Hierarchy by Pearl (2018):

Level 1: Association *P*(*y*| *x*) with the typical activity of “seeing” and questions including “How would seeing X change my belief in Y?”, in our use‐case above this was the question of “what does a feature in a histology slide the pathologist about a disease?”

Level 2: Intervention *P*(*y*| *do*(*x*), *z*) with the typical activity of “doing” and questions including “What if I do X?”, in our use‐case above this was the question of “what if the medical professional recommends treatment X—will the patient be cured?”

Level 3: Counterfactuals *P*(*y*_*x*_| *x*^′^, *y*^′^) with the typical activity of “retrospection” and questions including “Was Y the cause for X?”, in our use‐case above this was the question of “was it the treatment that cured the patient?”

For each of these levels we have to develop methods to measure effectiveness (does an explanation describe a statement with an adequate level of detail), efficiency (is this done with a minimum of time and effort) and user satisfaction (how satisfactory was the explanation for the decision making process). Again we should mention that there are three types of explanations: (1) a peer‐to‐peer explanation as it is carried out among physicians during medical reporting; (2) an educational explanation as it is carried out between teachers and students; (3) A scientific explanation in the strict sense of science theory (Popper, 1935). We emphasize that in this article we always refer to the first type of explanation.

## 5 CONCLUSION

AI is already one of the key technologies in our economy. It will bring changes similar to the introduction of the steam engine or electricity. However, concerns about potential loss of control in the Human‐AI relationship are growing. Issues such as autonomous driving and the unclear decision making of the vehicle, for example, in extreme cases shortly before an accident collision, have long been the subject of public debate. The same goes for the question of the extent to which AI can or should support medical decisions or even make them itself. In many cases it will be necessary to understand how a machine decision was made and to assess the quality of the explanation.

While rule‐based solutions of the early AI in the 1950s represented comprehensible “glass box” approaches, their weakness lay in dealing with uncertainties of the real world. Many problems from our everyday lives cannot be represented by formal, mathematical rules of logic. The failure of such algorithms to solve problems that are relatively simple for humans, such as natural language, recognizing faces, or understanding a joke, ultimately led to the “AI winter” in the 1980s. Only through the triumph of probabilistic and statistical learning methods in connection with the success of artificial neural networks (“deep learning”) did AI applications become increasingly successful.

Today, DL algorithms are very useful in our daily lives: autonomous driving, face recognition, speech understanding, recommendation systems, etc. already work very well. However, it is very difficult for people to understand how these algorithms come to a decision. Ultimately, these are so‐called “black box” models. The problem is that even if we understand the underlying mathematical principles and theories, such models lack an explicit declarative representation of knowledge. Early AI solutions (at that time called expert systems) had the goal from the beginning of making solutions comprehensible, understandable and thus explainable, which was also possible in very narrowly defined problems. Of course, we should mention that many problems do possibly not need explanations for everything at any time.

Here, the area of explainable AI is not only useful and necessary, but also represents a huge opportunity for AI solutions in general. The generally accused opacity of AI can thus be reduced and necessary trust built up. Exactly this can promote the acceptance with future users lastingly.

The main problem of the most successful current ML systems, recently emphasized by Pearl (2018), is that they work on a statistical, or model‐free mode, which entails severe limitations on their performance. Such systems are not able to understand the context, hence cannot reason about interventions and retrospection. However, such approaches needs the guidance of a **human model** similar to the ones used in causality research (Pearl, 2009; Pearl & Mackenzie, 2018) to answer the question “Why?”. The establishment of causability as a solid scientific field can help here.

“*Data can tell you that the people who took a medicine recovered faster than those who did not take it, but they cant tell you why. Maybe those who took the medicine did so because they could afford it and would have recovered just as fast without it*.”

Judea Pearl (2018), The Book of Why: The New Science of Cause and Effect

## CONFLICT OF INTEREST

The authors have declared no conflicts of interest for this article.

## ENDNOTE

1 Presented as a poster during the ICML 2009 workshop on Learning Feature Hierarchies, http://www.cs.toronto.edu/rsalakhu/deeplearning/program.html.
